# Loss of Infectivity of Influenza Virus and SARS-CoV‑2 during Aerosol Sampling

**DOI:** 10.1021/acs.estlett.6c00020

**Published:** 2026-02-13

**Authors:** Jin Pan, Nisha K. Duggal, Seema S. Lakdawala, Meher Sethi, Nahara Vargas-Maldonado, Vedhika Raghunathan, Anice C. Lowen, Linsey C. Marr

**Affiliations:** † Department of Civil and Environmental Engineering, 1757 Virginia Tech, Blacksburg, Virginia 24061, United States; ‡ Department of Occupational and Environmental Health, 4083 University of Iowa,Iowa City, Iowa 52242, United States; § Department of Biomedical Sciences and Pathobiology, Virginia-Maryland College of Veterinary Medicine, Blacksburg, Virginia 24061, United States; ∥ Department of Microbiology and Molecular Genetics, School of Medicine University of Pittsburgh, Pittsburgh, Pennsylvania 15219, United States; ⊥ Department of Microbiology and Immunology, Emory University School of Medicine,Atlanta, Georgia 30322, United States

**Keywords:** Influenza virus, aerosol sampling, infectivity, inactivation, bioaerosols

## Abstract

Our understanding of transmission of influenza virus and other respiratory viruses is limited by the difficulty of detecting infectious viruses in aerosol particles. Most aerosol sampling methods are believed to contribute to virus inactivation, but the magnitude of this sampling artifact is unknown. To investigate this question, we aerosolized influenza A virus (IAV) and SARS-CoV-2 suspended in human saliva into a small chamber (3.7 L). Aerosols settled for 10 min onto either cells or a thin layer of liquid medium that was immediately transferred to cells for plaque assay. Aerosols that deposited directly onto cells led to the formation of 100× more plaque forming units (PFU) compared to aerosols that deposited first into liquid medium. Further experiments ruled out uneven aerosol distribution in the chamber or inefficient virus recovery as causes of this discrepancy. These findings indicate that aerosolized IAV and SARS-CoV-2 lost infectivity by approximately 2 log_10_ PFU within ∼10 min unless they attached to cells quickly. As natural infection via inhalation occurs by direct deposition of the virus onto cells, we hypothesize that sampling directly onto cells more accurately reflects the potential for exposure to lead to infection.

## Graphical abstract

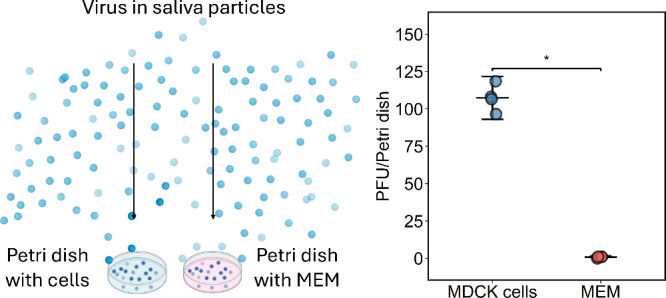

## Introduction

Influenza virus, SARS-CoV-2, and many other respiratory viruses are transmitted via respiratory aerosols and droplets.^1,2^ Aerosol sampling is a valuable tool for gaining insight into airborne transmission and improving the surveillance and management of respiratory viruses.^3–6^ Numerous studies have reported detection of viral RNA in aerosols in a variety of settings.^3,6–12^ However, RNA is not equated with infectivity, which is a metric of greater interest.

Many studies have attempted to culture influenza virus and SARS-CoV-2 in aerosol samples collected directly from exhaled breath or indoor air, and a subset of these has succeeded.^3,8,13–21^ Detecting infectious viruses in indoor air is difficult because of the large volume of air that must be sampled to overcome dilute concentrations of virus and the related challenge of maintaining virus viability during sampling. Using traditional techniques that collect aerosol particles onto a dry substrate, Xie et al.^3^ and Santarpia et al.^22^ found replication-competent influenza virus and SARS-CoV-2 in samples collected in a university and hospital, respectively. Their studies used either the NIOSH BC251 bioaerosol sampler^3^ or the Sartorius MD8 sampler^22^ equipped with gelatin filters. In general, researchers have found greater success detecting infectious virus using a condensation sampling method that deposits particles into liquid.^8,21,23,24^ This approach is considered to be gentler than traditional aerosol sampling techniques, hinting that sampling artifacts may lead to virus inactivation and thus underestimation of the potential for airborne virus transmission.

Traditional bioaerosol sampling usually employs filters, impactors, or impingers.^25–28^ If the sample is collected onto a solid substrate, it is then transferred to a liquid prior to analysis. Several studies have assessed the loss of infectivity of influenza virus^29^ or SARS-CoV-2 ^30–32^ associated with aerosol sampling under laboratory-controlled conditions. Some studies reported no loss in infectivity of aerosolized virus when using the SKC Biosampler^29,30^ or polytetrafluoroethylene (PTFE) filters,^30^ whereas others observed a 60–70% decrease in infectivity during 10 min of sampling onto PTFE filters,^31^ or even an immediate loss of 50–60% upon aerosolization at 40% relative humidity (RH).^32^

Loss of infectious virus in aerosols can take place at three different stages: aerosolization, aging in the air, and sample collection and processing.^33,34^ At each stage, both physical and biological losses may occur. Physical loss refers to removal of a particle by, for example, gravitational deposition, whereas biological loss refers to virus inactivation, even if the particle is still physically present.^33^ These losses can be difficult to quantify accurately.

Focusing on biological losses during sample collection and processing, we investigated the loss of infectivity of aerosolized influenza A virus (IAV) and SARS-CoV-2 that deposited directly onto cells compared to aerosolized virus that deposited into liquid media. The former approach mimics the transport of virus-laden aerosols to respiratory cells, whereas the latter is a common step in aerosol sampling or processing. Relying on passive sampling and forgoing use of an air sampler avoid losses that may be introduced by sampling and elution. We observed substantial loss of virus infectivity in aerosols that were deposited into liquid media compared to those that deposited directly onto cells. By revealing challenges in airborne virus detection, our findings will enhance the interpretation of air sampling results and foster improvements in sampling strategies.

## Methods and Materials

### Cells and Virus Preparation

For influenza A virus, we cultured and maintained Madin-Darby Canine Kidney (MDCK) cells (ATCC, CCL-34) at 37 °C and 5% CO_2_ in cell culture media, which consisted of 1× Minimum Essential Medium (MEM) (Gibco, 12360038), 1% (v/v) l-glutamine (Gibco, 25030081), 10% gamma irradiated fetal bovine serum (VWR, 97068–086), and 1% penicillin-streptomycin (Gibco, 15140122). We passaged influenza A/California/07/2009 virus in infection media on confluent MDCK cells, which contained 1× MEM, 1% l-glutamine, 1% antibiotic-antimycotic (Gibco, 15240062), and 1 μg/mL TPCK trypsin (Thermo Fisher, 20233). We harvested virus in the supernatant after centrifugation at 200 × g for 10 min.

For SARS-CoV-2, we cultured and maintained Vero E6 TMPRSS2 ACE2 cells (BEI, NR-54970) at 37 °C and 5% CO_2_ in cell culture media, which consisted of Dulbecco’s modified Eagle medium (DMEM) (Corning, 10–017-CV), 5% gamma-irradiated fetal bovine serum (FBS) (VWR, 97068–086), 100 units/ml penicillin, and 100 μg/mL streptomycin (Gibco, 15140122). We passaged the SARS-CoV-2 strain USA-WA1/2020 (BEI, NR-52281) on confluent Vero E6 TMPRSS2 ACE2 cells. We harvested virus in the supernatant and removed extracellular materials after centrifugation at 200 × g for 10 min.

Virus stocks were aliquoted and stored at −80 °C.

### Aerosolization and Collection of Virus

We aerosolized IAV or SARS-CoV-2 and collected samples into a small chamber used in a previous study^35^ (Figure 1). The chamber (Stoelting Company, 53917) had dimensions of 23 cm (L) × 12.7 cm (W) and 12.7 cm (H) and had a sliding top. We added ports to it on one side. A medical, jet-style nebulizer (Philips Respironics) loaded with 2 mL of 10^5^ PFU/mL of IAV or SARS-CoV-2 suspended in pooled human saliva (Innovative Research, 33265) delivered aerosolized virus into the chamber at a flow rate of ∼5 LPM. We installed a small fan in front of the aerosol inlet to ensure thorough mixing of aerosols in the chamber. We placed four 35 mm polystyrene Petri dishes (Sigma-Aldrich, CLS430588–500EA) on the bottom of the chamber. Two of the Petri dishes contained confluent MDCK cells (for IAV) or Vero E6 TMPRSS2 ACE2 cells (for SARS-CoV-2) with 200 μL of phosphate-buffered saline (PBS) added on top to prevent drying. The other two dishes contained 700 μL of MEM, an amount selected to cover the entire bottom of the Petri dish. We attached a high-efficiency particulate air (HEPA) filter capsule (TSI, 1602345) and a pump (SKC, AirChek XR5000) running at 5 LPM to the outlet of the chamber to collect all remaining aerosols at the end of each experiment for the purposes of enhancing safety and minimizing cross-contamination. We measured the RH in the chamber using a HOBO logger (Onset, UX100-011) as shown in Figure S2. Figure 1 is a schematic of the experimental setup.

**Figure 1:**
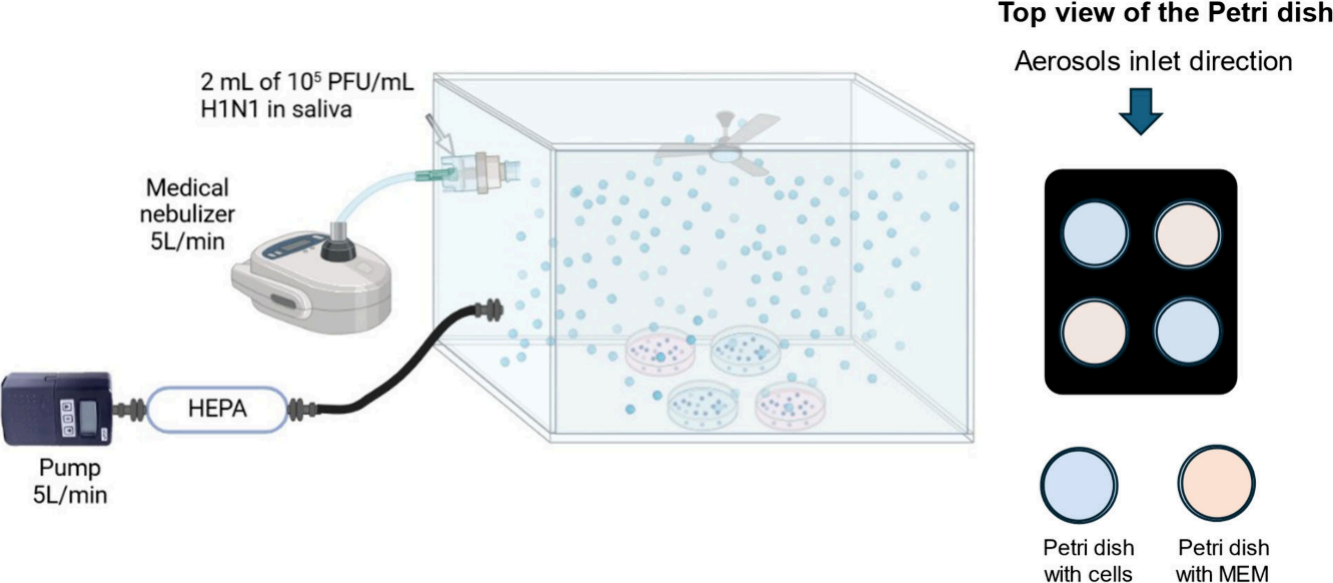
Schematic of the experimental setup for aerosol generation and virus collection. Petri dishes with cells are shown in blue, and those with MEM are shown in pink. The top of the chamber has a small slit, which is not shown in the figure. The figure was generated at Biorender.com.

All experiments took place inside a biosafety cabinet. To fill the chamber with aerosolized virus, we ran both the nebulizer and the fan for 2 min. Excess air was vented through a small slit at the top of the chamber. After 2 min, we turned off both the nebulizer and the fan, allowing aerosols to settle onto the Petri dishes and surrounding floor of the chamber for 10 min. The mode diameter of these polydisperse aerosols was approximately 1.3 μm, as measured by an Aerodynamic Particle Sizer (TSI Inc., APS 3321) that can detect particles of aerodynamic diameter 0.5–20 μm. Almost all particles larger than 1.8 μm should have settled to the chamber floor within 10 min (Figure S3), and lesser fractions of smaller particles also would have settled during this period. We then turned on the pump for 2 min to remove the remaining aerosols from the chamber, with makeup air entering through the slit at the top. Finally, we opened the chamber and removed the Petri dishes to analyze them by plaque assay.

### Quantification of Infectious Virus and Recovery Efficiency

To quantify infectious IAV or SARS-CoV-2 that deposited on Petri dishes with cells, we incubated the Petri dishes at 37 °C and 5% CO_2_ upon their removal from the chamber for 1 h. After incubation, we added 2 mL of agarose overlay (composition found in SI) to each dish and incubated it for another 48 h. For Petri dishes containing 700 μL of MEM, immediately upon removal from the chamber, we divided the entire volume into three portions, each measuring 200–300 μL, and transferred them to three wells of a six-well plate with confluent MDCK or Vero E6 TMPRSS2 ACE2 cells. We then followed the same procedures as for the Petri dishes with cells. We counted the number of plaques after incubation for 48 h of incubation.

We determined the recovery efficiency from media in Petri dishes by spiking 1 μL of virus stock with known titer into Petri dishes containing 1000 μL of MEM alone, followed by pipetting up and down for thorough mixing. Then we withdrew 300 μL for titering. These plates then went through the entire aerosolization (including virus in saliva), deposition, and quantification process. We compared the titer recovered from the media to the spiked titer after subtracting the titer of virus that deposited from the air, to determine the recovery efficiency. We also used a fluorescent tracer to estimate the volume of saliva that deposited in each dish (Figure S1).

### Statistics

We performed a Brunner–Munzel test that allows for ties in data to compare the number of PFU deposited on Petri dishes containing cells and those with MEM. We used R 4.5.2. A *p*-value of <0.05 was considered statistically significant.

## Results and Discussion

### Deposition Directly onto Cells Better Preserved Infectivity

Aerosolized IAV or SARS-CoV-2 was allowed to deposit onto cells in Petri dishes or MEM in Petri dishes for 10 min. Figure 2 shows that we consistently recovered ∼ 100 PFU from dishes with cells but only 0–2 PFU from dishes with MEM. Since we added PBS on top of the cells to prevent drying, we repeated the experiments with IAV using PBS instead of MEM in the Petri dishes without cells to evaluate the impact of the media. We recovered up to 80 PFU from dishes containing cells, compared to <1 PFU from those containing PBS (Table S1). This finding indicated that the type of media did not contribute to the large differences observed between Petri dishes with cells and those with media only.

**Figure 2:**
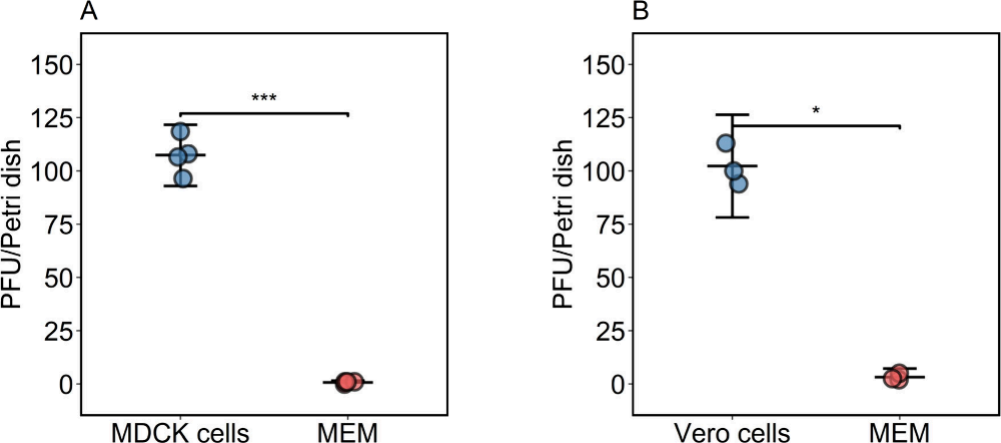
Number of plaque-forming units (PFU) of influenza A virus (A) or SARS-CoV-2 (B) recovered from Petri dishes with cells (blue) or with MEM (red) after aerosolized virus was deposited for ∼10 min. Each dot represents an independent replicate, calculated as the average of two technical replicates. Error bars represent standard deviations, and the middle bars represent means. * *p* < 0.05, according to the Brunner–Munzel test.

To determine whether the observed differences were due to uneven spatial deposition of aerosols inside the chamber, we placed Petri dishes with MDCK cells at all four locations (Figure 1, Figure S1) and quantified the infectious virus after 10 min of deposition. The amount of infectious IAV and the volume of deposited saliva were similar across all Petri dishes (Figure S1), indicating that aerosols were evenly distributed and deposited.

We further evaluated the virus recovery efficiency from Petri dishes containing MEM by spiking them with a known titer of IAV and subjecting them to the full aerosolization, deposition, and recovery process. The amount of virus spiked into the dishes was 20–40× larger than the amount recovered in the aerosolization experiments. No significant differences were observed between the spiked and recovered titers (Table S2), suggesting that we recovered nearly 100% of the spiked virus. The high recovery efficiency also suggested that the spiked IAV did not lose infectivity or decay in MEM over the course of the experiment. This result further ruled out the possibility that poor recovery or rapid decay caused by MEM contributed to the differences between Petri dishes with cells and those with MEM only. In summary, we detected ∼100× more infectious IAV or SARS-CoV-2 in aerosols that deposited directly on cells rather than into MEM, implying the virus lost infectivity by ∼2 log_10_ PFU unless it attached to cells quickly.

### Interpretation of Rapid Loss of Infectivity of Aerosolized Viruses

Other studies that have investigated the loss of virus infectivity soon after aerosolization provide context for our results. Fabian et al. found no reduction in terms of the ratio of total to infectious influenza virus particles aerosolized by a Collison nebulizer and collected by an SKC BioSampler during a 7.6 s period between aerosol generation and collection.^29^ Ratnesar-Shumate et al. reported no loss of infectivity in airborne SARS-CoV-2 compared to the theoretical maximum using an SKC BioSampler or PTFE filters when the aerosols were collected immediately after generation for 3, 13, or 33 min.^30^ However, Dabisch et al. observed ∼0.5 log_10_ decay in infectivity of SARS-CoV-2 in aerosols that were collected on PTFE filters for 10 min, compared to the theoretical maximum and results with an impinger (SKC Inc., 225–0020).^31^ In summary, previous studies observed zero to 0.5 log_10_ decay of aerosolized virus upon collection. Yet, in most cases, the actual titers were compared to a theoretical maximum, whose calculation may involve uncertainties tied to assumptions. Allowing aerosols to settle directly on cells or media, as in our study, removed the uncertainties introduced by sampling and recovery.

We are aware of two other research groups that have collected virus-laden aerosols directly onto cells. Shankar et al. used a 3-stage BioCascade impactor (size cuts: >9.43 μm, 3.81–9.43 μm, 1.41–3.81 μm) to collect aerosolized human coronavirus (HCoV)-OC43 directly onto cells.^36^ They successfully recovered >95% of viable virus on each stage. Although they did not compare deposition onto cells against deposition into medium, their results indicated that direct inoculation of aerosolized virus onto cells may preserve viral infectivity well. In a study of respiratory particles emitted by infected individuals, Vargas-Maldonado et al. discovered that aerosol samplers collected less infectious IAV than was obtained by directly depositing larger particles onto cell-culture plates.^37^ This was likely due to both the limited efficiency of the aerosol sampling methods and the small amount of infectious IAV present in the small aerosols.

Our experimental design controlled for losses during aerosolization and aging in the air, as these would be the same for both treatments, so differences in results can be isolated to the period after virus deposition onto the liquid layer with or without cells underneath it. We expect that virion movement in liquid was primarily driven by diffusion rather than gravitational settling, with a characteristic diffusion time of less than 2 min for virions to attach to cells in Petri dishes containing cells (detailed calculations in SI). The handling procedures for transferring and titering viruses after deposition into media should not result in virus decay, as demonstrated by our recovery test (Table S2). Therefore, the lack of detection of infectious IAV or SARS-CoV-2 in these samples appears to be due to rapid inactivation of virus after it deposited from the air, while it was in the liquid. Aerosolization and aging may have initiated the inactivation process and damaged virions, such that if they did not attach to cells rapidly, they lost infectivity and could not be detected by downstream infectivity assays. It is possible that virions were aggregated in aerosols and that these aggregates disassembled after deposition into liquid. Aggregation may increase viral infectivity by enabling cellular coinfection^38,39^ and may stabilize virus against environmental stressors and disinfectants, prolonging survival.^40^ However, there is no direct evidence of viral aggregation in aerosols; additional research is needed to test such a hypothesis.

### Limitations and Implications

Our study showed that collection of aerosolized IAV and SARS-CoV-2 directly onto cells allowed for detection of ∼100× more infectious virus compared to collection into media. Limitations include the use of MDCK and Vero E6 TMPRSS2 ACE2 cells, which differ from human respiratory cells. Additionally, we aerosolized the virus using a jet nebulizer, which produces aerosols through a mechanism different from that of natural respiratory aerosols. The differences in aerosolization mechanisms may affect virus integrity and aggregation, which remain to be investigated. Therefore, we should be cautious about extending these findings to all aerosol sampling scenarios. We did not analyze viral RNA in Petri dishes with MEM or cells; thereby, we cannot exclude the possibility of poor recovery due to virions binding to polystyrene dishes. However, most virions are expected to remain suspended in the liquid (calculations in SI) and are unlikely to reach the bottom surface of the dishes. We also expect any binding to be minimal because prior studies have shown good recovery of influenza virus, in terms of gene copies, from plastic materials.^41–43^ In addition, quantifying viral RNA in Petri dishes with cells to control for deposition would be technically challenging due to rapid virus–cell binding.

The findings of our study suggest that aerosolized IAV and SARS-CoV-2 lose infectivity if they do not attach to cells within a short time frame. This suggests that mechanically aerosolized IAV and SARS-CoV-2 are fragile and may quickly lose their infectivity. Additionally, it emphasizes that environmental sampling of aerosols, which often includes extended periods of collection, transport, and processing, may underestimate the infectivity of viruses in aerosols. Thus, exposure to infectious IAV or SARS-CoV-2 could be significantly higher than suggested by traditional methods of air sampling, as aerosolized viruses have the potential to attach directly to respiratory cells upon inhalation.

## Supplementary Material



## Notes

Details of plaque assay overlay composition; infectivity of aerosolized virus after deposition into PBS; spatial distribution of deposited virus-laden aerosols and deposited volume of saliva; relative humidity and temperature in the chamber; measured initial number of aerosol particles in column of air above a Petri dish and the estimated deposited number of particles on a Petri dish; calculation of virion movement in liquid; comparison to previous literature on virus inactivation on surfaces (PDF)
