# Supplementary material for: Loss of Infectivity of Influenza Virus and SARS-CoV‑2 during Aerosol Sampling
Source: Environ Sci Technol Lett. 2026 Feb 13;13(3):380–5. doi: 10.1021/acs.estlett.6c00020 (PMC12980836; doi:10.1021/acs.estlett.6c00020)
Supplement: Supplementary file 1 [file ez6c00020_si_001.pdf]

Supporting Information

Loss of infectivity of influenza virus and SARS-CoV-2 during aerosol sampling

Jin Pan[1, 2, *], Nisha K. Duggal[3], Seema S. Lakdawala[4, 5], Meher Sethi[5], Nahara Vargas-Maldonado[5], Vedhika Raghunathan[5], Anice C. Lowen[5], Linsey C. Marr[1]

[1] Department of Civil and Environmental Engineering, Virginia Tech, Blacksburg, VA, USA 24061

[2] Department of Occupational and Environmental Health, University of Iowa, Iowa City, IA, USA 52242

[3] Department of Biomedical Sciences and Pathobiology, Virginia-Maryland College of Veterinary Medicine, Blacksburg, VA, USA 24061

[4] Department of Microbiology and Molecular Genetics, University of Pittsburgh School of Medicine, Pittsburgh, PA, USA 15219

[5] Department of Microbiology and Immunology, Emory University School of Medicine, Atlanta, GA, USA 30322

*Corresponding author: Jin Pan, jin-pan@uiowa.edu

Summary of content

This supporting information document contains the following information:

- Details of plaque assay overlay composition
- Infectivity of aerosolized virus after deposition into PBS
- Efficiency of virus recovery
- Spatial distribution of deposited virus-laden aerosols and saliva volume
- Relative humidity and temperature in the chamber
- Measured initial number of aerosol particles in column of air above a Petri dish and the estimated deposited number of particles on a Petri dish
- Calculation of virion movement in liquid
- Comparison to previous literature on virus inactivation on surfaces

Number of pages: 11

Number of figures: 3

Number of tables: 2

Overlay composition

For IAV, the overlay consisted of 10% 10×MEM (Gibco, 11430030), 1% L-glutamine (Gibco, 25030081), 2% of 7.5% sodium bicarbonate (Gibco, 25080094), 1.5% HEPES buffer (Gibco, 15630080), 1% Penicillin-Streptomycin (Pen/Strep) (Gibco, 15140122), 0.01% of 1% Diethylaminoethyl dextran (DEAE-dextran) (Thermo fisher, J63781.14), 1 μg/mL TPCK trypsin (Thermo Fisher, 20233), 2.1 mg/mL bovine serum albumin (Fisher Scientific, SH3057402), and 0.64% agarose (SeaKem LE Agarose).

For SARS-CoV-2, the overlay consisted of 0.8% agarose and Ye-Lah medium.[1]

Infectivity of virus deposited into PBS

Since we used PBS in Petri dishes with cells and MEM in Petri dishes with media alone, we compared MEM and PBS to investigate whether the type of media led to differences between Petri dishes with cells and with media only. We added 700 µL PBS instead of MEM to the Petri dishes with media and kept the Petri dishes with cells the same as described in main text (i.e., cells topped with 200 µL of PBS). All Petri dishes then went through the full course of aerosolization and deposition. Table S1 shows that the number of infectious IAV was significantly higher on Petri dishes containing cells compared to those with PBS, as observed similarly with MEM.

We also analyzed the infectious titer if we increased the volume of PBS on cells from 200 µL to 700 µL. Although cytopathic effects were observed, quantification of the titer was challenging due to plaque clustering. We cannot determine if the titer was higher or lower with 700 µL compared to 200 µL, but higher volume appeared to reduce cytopathic effects.

Table S1. Comparison between the amount of infectious IAV recovered from Petri dishes with cells and Petri dishes with PBS, in terms of number of plaques.

Independent replicate	Number of plaques on Petri dishes with cells	Number of plaques on Petri dishes with PBS
1	80	0
2	76	0.5

Efficiency of virus recovery

We evaluated virus recovery efficiency from Petri dishes containing MEM by spiking them with a known titer of IAV and subjecting them to the full aerosolization, deposition, and recovery process. The amount of virus spiked into the dishes was 20-40× larger than the amount recovered in the aerosolization experiments. No significant differences were observed between the spiked and recovered titers (Table S2).

Table S2. Two independent replicates comparing the spiked titer to the recovered titer of infectious IAV (after subtracting the titer of virus that deposited from the air) from Petri dishes containing 700 µL of MEM. The spiked and recovered titers were not significantly different ($p > 0.05$) according to the Brunner-Munzel test.

Replicate	Spiked titer (PFU/Petri dish)	Recovered titer (PFU/Petri dish)
1	45.5	43
2	45.5	44

Spatial distribution of deposited virus-laden aerosols and deposited saliva volume in each Petri dish

In order to validate that the differences between Petri dishes with MDCK cells or with MEM were not due to uneven distribution of deposited virus-laden aerosols, we placed identical Petri dishes with cells in all four positions, as shown in Figure S1. After aerosolization and deposition, the amount of infectious IAV in terms of plaques was similar at all four positions (Figure S1). We also analyzed the saliva volume deposited in each Petri dish by adding disodium fluorescein (Ex: 495 nm, Em: 520 nm) into pooled human saliva at a final concentration of 1 µg/mL. Following the procedures described in the main text, we aerosolized fluorescent saliva aerosols and allowed them to deposit onto four Petri dishes containing PBS only. We prepared a standard curve by serially diluting fluorescent saliva in PBS, with dilution factors from 1 to 10^{-4}. The fluorescent signal strength of the solution in PBS-containing Petri dishes after saliva deposition was measured using a plate reader (BioTek Synergy HTX) and compared to the standard curve to estimate the deposited volume. The deposited saliva volume was similar across all four locations. If the viral concentration in aerosols were the same as that in bulk (10^5 PFU/mL), the number of infectious virions expected to deposit on each Petri dish would be 200-300 PFU. Therefore, our observations of around 100 PFU are reasonable when taking into account virus inactivation resulting from aerosolization and uncertainties about the distribution of virions among aerosol particles of different sizes.

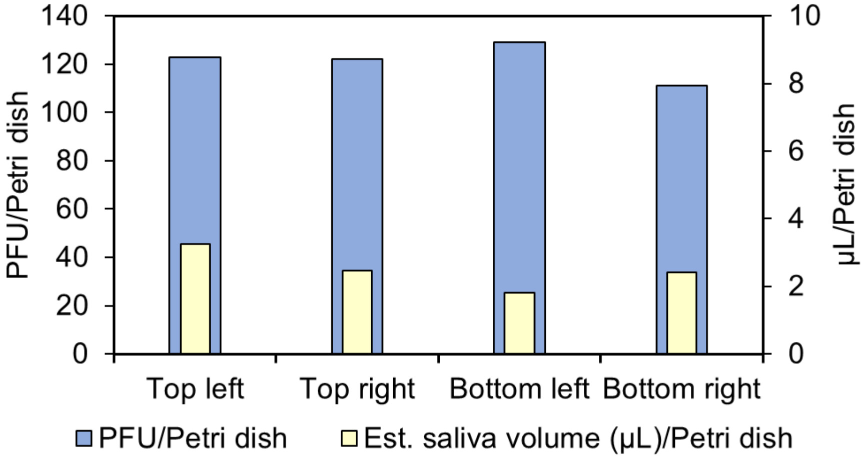

Figure S1. Number of plaques recovered from Petri dish with cells and the estimated volume of saliva deposited at four locations in the chamber.

91 **Relative humidity (RH) and temperature in the chamber**

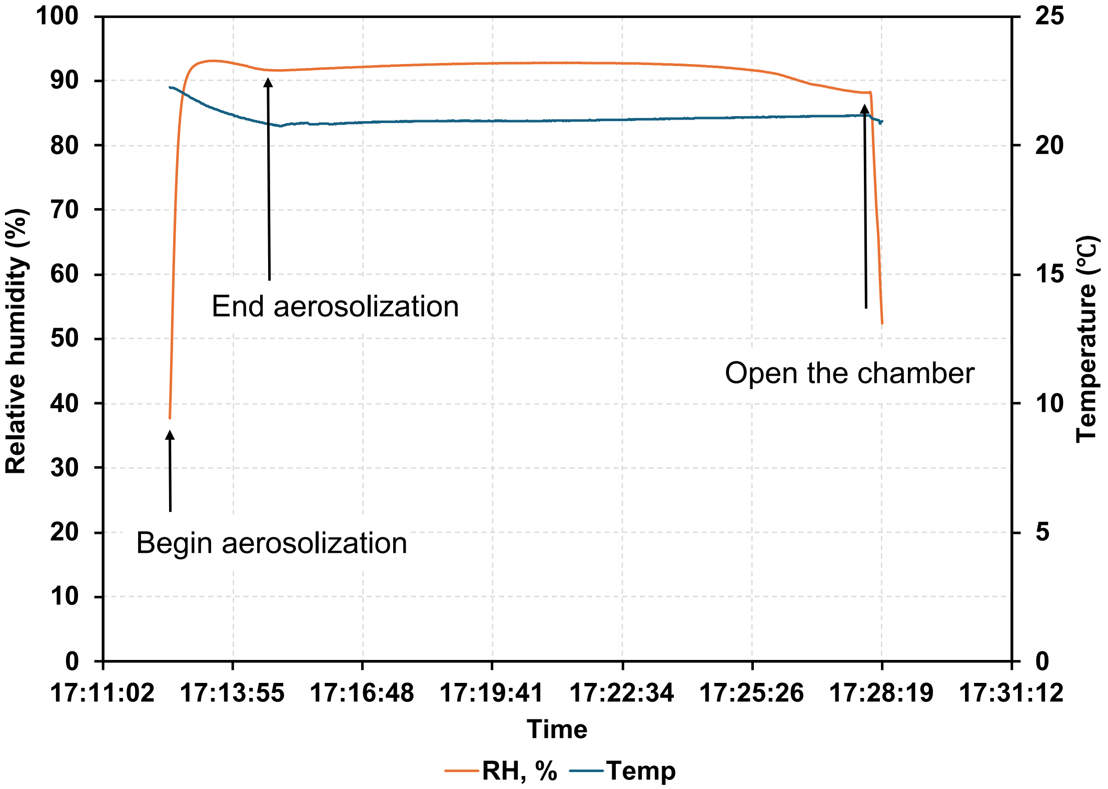

92

93 Figure S2. RH and temperature in the chamber during a representative experiment.

94

Estimated aerosol deposition on one Petri dish

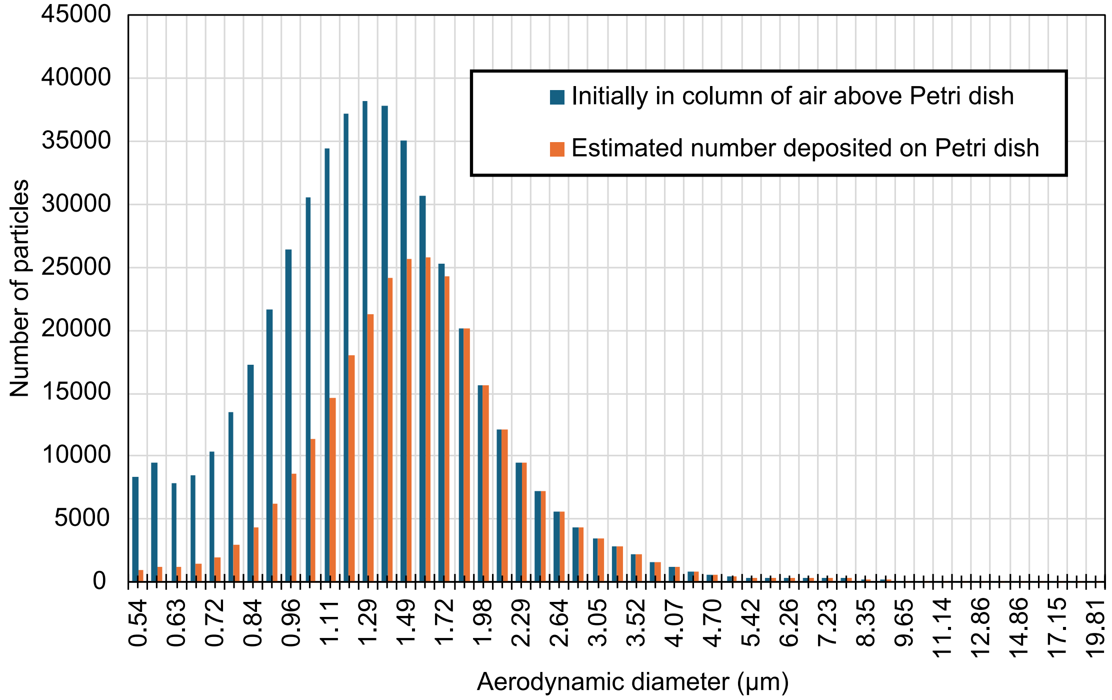

Figure S3. Number of particles, determined by APS, initially in the column of air above one Petri dish and the number predicted to deposit after 10 min. We did not attempt a similar analysis for PFU, based on the aerosol size distribution, because some particles outside the APS' range of 0.5-20 µm were likely present, and some would have deposited before acquisition of the APS measurement.

Movement of virions in liquid

We estimated the characteristic diffusion time for virions to attach to cells after they deposited into the liquid layer on top of cells. We added 200 μL of PBS on top of cells in a 35-mm Petri dish, which has an area of 9 cm^2. Below are the parameters and assumptions necessary to conduct the calculations.

1) With cells beneath the liquid, the liquid layer was not a flat surface. Instead, surface tension causes the liquid to stretch and curve, leading to accumulation toward the edges of the Petri dish. We assumed ~90% of liquid volume was around the edge while only 10% of liquid volume was in the middle of the Petri dish. Therefore, the depth of the liquid layer of 200-μL PBS in a 35-mm Petri dish in the presence of cells was approximately:

$$l = \frac{10\% \times 200\ \mu L}{9\ cm^2} = 22\ \mu m$$

2) The diffusion coefficient, D, of virions in water is estimated to be 15.5 $\mu m^2/s$.[2] This estimation was based on Bromegrass mosaic virus diffusing in water at 20°C, limited by the data availability. Therefore, the characteristic time for virions to diffuse through the liquid layer to attach to cells was around:

$$t = \frac{l^2}{D} = \frac{(22\ \mu m)^2}{15.5\ \ \mu m^2/s} = 31.2\ s$$

3) We also estimated the diffusion coefficient, D, using the Stokes-Einstein equation, assuming virions are spheres diffusing in water. The virion diameter, d, is 100 nm. The Boltzmann constant, k_B, is 1.38×10^{-23} $J\cdot K^{-1}$. The absolute temperature, T, is 293 K (20°C). The viscosity of water, μ_w, is 1.003×10^{-3} Pa·s at 20°C.

$$D = \frac{k_B T}{3\pi\mu_w d} = \frac{1.38 \times 10^{-23} \cdot 293}{3\pi \cdot 1.003 \times 10^{-3} \cdot 100 \times 10^{-9}} \approx 4.3\ \mu m^2/s$$

This value is smaller than the value reported in previous literature,[2] resulting in a characteristic time of diffusion to be 112.6 s, or less than 2 min.

The characteristic time of diffusion is an average that may overestimate the actual diffusion time for some virions to attach to cells and underestimate the actual time for others. Since the cells do not form a perfectly smooth and uniform surface, it is likely that some cells protruded more than others, resulting in variations in liquid layer depth across the surface.

For comparison, we compared the characteristic diffusion time to the time for virions to settle through the liquid layer by gravity to identify which mechanism dominated the movement of virions in liquid. Below are the parameters and assumptions:

1) We assumed that once virions deposited in liquid, they behaved as individual spherical particles 100 nm in diameter. In reality, virions are not perfect spheres. They may also cluster due to binding to respiratory proteins. We made the assumption to simplify the calculation.
2) We assumed that the viscosity of liquid (PBS or MEM) is similar to that of water, μ_w, 1.003×10^{-3} Pa·s at 20°C based on previous literature.[3]
3) We assumed that the movement of virions in liquid follows Stoke's law, as verified by Reynold's number, Re < 1.
4) We ignored the deceleration period when virions transitioned from air to liquid, assuming that this period was short compared to settling time due to a much higher viscosity of liquid compared to air. We thus assumed the force equilibrium among buoyance, drag force, and gravity, was established instantly.
5) We assumed the density of virions, ρ_v, to be 1.4 g/mL, based on data for rhinovirus.[4] The density of water, ρ_w, is 1.0 g/mL. The diameter of virions, d_v, is 100 nm.

With the above assumptions, we estimated the settling velocity following Stoke's law as below.

$$v = \frac{(\rho_v - \rho_w) \cdot d_v^2 \cdot g}{18\mu_w} \approx 2.2 \times 10^{-3}\ \mu m/s$$

The characteristic time for virions to settle through the liquid layer depth, l, which was 22 μm as calculated above, was:

$$t = \frac{l}{v} = 10^4\ s$$

Gravitational settling of virions was much slower than diffusion. Therefore, the movement of virions in liquid was dominated by diffusion.

Comparison to previous literature on virus inactivation on surfaces

We compared our results to previous literature that investigated virus inactivation in aerosols after deposition on surfaces. Fedorenko et al. observed that phi6, an enveloped bacteriophage commonly used as a surrogate for IAV or SARS-CoV-2, exhibited decay of at least 2 $\log_{10}$ PFU/mL compared to bulk levels when suspended in water or SM buffer and sprayed onto Petri dishes across all RHs.[5] When phi6 was suspended in saliva, infectivity decayed by approximately 1.5 $\log_{10}$ PFU/mL at 57% RH and less than 1 $\log_{10}$ PFU/mL at other RHs.[5] The researchers' approach did not differentiate among aerosolization, aging, and sample collection and processing, because the reference for comparison was virus in bulk suspension. Thus, the reported decay represented the total loss of infectious virus across all three stages. Parry-Nweye et al. reported that the titer at 25% RH was at least 2 $\log_{10}$ PFU/mL greater than at 45% RH after aerosolized phi6 suspended in Tryptic-Soy Broth had settled on various surfaces.[6] They did not specify a baseline for comparison in this scenario but instead compared the titer measured at two different RH levels. Oswin et al. reported an immediate loss of SARS-CoV-2 infectivity by 50%-60% when the virus was suspended in MEM with 2% FBS and then aerosolized at 40% RH compared to 90% RH;[7] the aerosols were collected in a Petri dish containing 5 to 10 mL of Dulbecco's Modified Eagle Medium (DMEM) with 2% FBS. Their approach minimized any physical losses during sample collection. It also minimized the influence of aging, as deposited aerosols were collected immediately. Additionally, samples collected at both 40% RH and 90% RH were subject to the same processing. Thus, their results suggest that more rapid inactivation of virus occurred at 40% RH compared to 90% RH. However, the difference with RH does not explain the results in our study, as the RH remained above 90% in the chamber upon aerosolization (Figure S2), and all Petri dishes, whether they contained cells or media, were exposed to the same conditions. Oswin et al. also reported that the infectivity of aerosolized SARS-CoV-2 decreased by 90% within 10 min at 90% RH when collected in media;[7] this trend was similar to our findings.

References

(1) Poole-Smith, B. K.; Hemme, R. R.; Delorey, M.; Felix, G.; Gonzalez, A. L.; Amador, M.; Hunsperger, E. A.; Barrera, R. Comparison of Vector Competence of Aedes mediovittatus and Aedes aegypti for Dengue Virus: Implications for Dengue Control in the Caribbean. *PLOS Neglected Tropical Diseases* **2015**, *9* (2), e0003462. DOI: 10.1371/journal.pntd.0003462.
(2) Bockstahler, L. E.; Kaesberg, P. The Molecular Weight and Other Biophysical Properties of Bromegrass Mosaic Virus. *Biophys J* **1962**, *2* (1), 1–9. DOI: 10.1016/s0006-3495(62)86836-2.
(3) Poon, C. Measuring the density and viscosity of culture media for optimized computational fluid dynamics analysis of in vitro devices. *Journal of the Mechanical Behavior of Biomedical Materials* **2022**, *126*, 105024. DOI: https://doi.org/10.1016/j.jmbbm.2021.105024.
(4) Dans, P. E.; Forsyth, B. R.; Chanock, R. M. Density of Infectious Virus and Complement-Fixing Antigens of Two Rhinovirus Strains. *Journal of Bacteriology* **1966**, *91* (4), 1605–1611. DOI: 10.1128/jb.91.4.1605-1611.1966.
(5) Fedorenko, A.; Grinberg, M.; Orevi, T.; Kashtan, N. Survival of the enveloped bacteriophage Phi6 (a surrogate for SARS-CoV-2) in evaporated saliva microdroplets deposited on glass surfaces. *Scientific reports* **2020**, *10* (1), 22419.
(6) Parry-Nweye, E.; Liu, Z.; Dhaouadi, Y.; Guo, X.; Huang, W.; Zhang, J.; Ren, D. Persistence of Phi6, a SARS-CoV-2 surrogate, in simulated indoor environments: Effects of humidity and material properties. *PLOS ONE* **2025**, *20* (1), e0313604. DOI: 10.1371/journal.pone.0313604.
(7) Oswin, H. P.; Haddrell, A. E.; Otero-Fernandez, M.; Mann, J. F.; Cogan, T. A.; Hilditch, T. G.; Tian, J.; Hardy, D. A.; Hill, D. J.; Finn, A. The dynamics of SARS-CoV-2 infectivity with changes in aerosol microenvironment. *Proceedings of the National Academy of Sciences* **2022**, *119* (27), e2200109119.
